# Bioinformatic and Metabolomic Analysis Reveal Intervention Effects of Chicory in a Quail Model of Hyperuricemia

**DOI:** 10.1155/2018/5730385

**Published:** 2018-12-03

**Authors:** Meng Bian, Zhijian Lin, Yu Wang, Bing Zhang, Gaoxi Li, Haige Wang

**Affiliations:** Department of Clinical Chinese Pharmacy, School of Chinese Pharmacy, Beijing University of Chinese Medicine, Beijing 100029, China

## Abstract

*Background*. Hyperuricemia (HUA) is a kind of a metabolic disease that seriously threatens human health worldwide. Chicory, a natural herbal medicine, has an obvious effect of reducing uric acid. The aim of this study is to explore the potential components and pharmacological pathways that may play a role in hypouricemia activity of chicory. Bioinformatics and metabonomics were applied to this research. Firstly, component-target network was used to identify possible components related to the pharmacological properties and their corresponding mechanisms pathway of chicory. Afterwards, animal pharmacodynamic experiments were performed. Blood and stool samples were collected for untargeted metabolomic analysis by dint of UHPLC-Q-TOF/MS methods, and principal component analysis (PCA) and partial least squares-discriminant analysis (PLS-DA) were performed for the pattern recognition and characteristic metabolites identification. Significant enriched function pathways were used in bioinformatics suggesting that chicory might have the effect of regulation of lipolysis in adipocytes. PLS-DA analysis was applied to discover differentiating metabolites, and pathway enrichment analysis indicated that chicory had powerful effects of glycosylphosphatidylinositol- (GPI-) anchor biosynthesis, inositol phosphate metabolism, glycerophospholipid metabolism, and steroid hormone biosynthesis. Combining bioinformatics and metabolomics results, we consider that chicory may develop on lowering uric acid by adjusting lipid metabolism. In addition, we chose quail as animal model innovatively and discussed the treatment of hyperuricemia with chicory in multiple methods, which may render reference for the research of HUA.

## 1. Introduction

Uric acid as the end-product of purine metabolism is due to the lack of uricase in human being and avian which causes the elevated uric acid level in serum [1]. Normal levels of uric acid may play a role in regulating the immune responses, accommodating antioxidant balance, and adjusting blood pressure [2]. However, the excessive level of uric acid is an independent risk factor for metabolic syndrome, cardiovascular disease, and kidney disease [3], even causing gout, urolithiasis, and urate nephropathy [4]. Either uric acid reabsorption increases, or excretion decreases, can lead to HUA. With the change of lifestyle and dietary habits, the incidence of HUA has been increasing year by year worldwide [5]. Thus, it is a vital clinic requirement to search safe and effective drugs for the intervention of HUA. In the treatment of HUA, uricostatic agent and uricosuric agent can effectively control the serum uric acid, yet the side effects in the clinic application cannot be ignored. Severe drug-induced hypersensitivity syndrome is the main side effect of allopurinol and benzbromarone [6, 7]. In recent years, the use of natural herbal medicine in the prevention and treatment of HUA has brought about the widespread attention; the advantages of herbal medicine are low incidence of adverse effects and remarkable curative effect.

*Cichorium intybus L.*, as we know as chicory, is a traditional herb from Asteraceae family. Chicory is used as a traditional Uighur folk medicine to treat icterohepatitis, with the function of clearing liver and cholagogue, strengthening stomach and promoting digestion. Literature has shown that the pharmacological actions of chicory include antioxidant, anti-inflammatory, antihypoglycemic, and antihyperlipidemic effects [8]. In previous studies, we found that chicory reduced the level of serum uric acid significantly, and the effect of lowing uric acid was mainly related to inhibiting the activity of xanthine oxidase, as well as promoting the expression of uric acid transporter ABCG2 and OAT3 mRNA in the intestine and kidney, respectively [9, 10]. However, it is not clear how the chicory alters the purine metabolism to reduce uric acid. What is more, chicory may have other unknown medicinal virtues yet to be discovered. Therefore, to find the possible antihyperuricemia mechanism of chicory, we predicted the components, targets, and pathways about pharmacological action of chicory by using network pharmacology. Hereafter, we chose quail, which is the same as human uric acid metabolism pathway, as an experimental animal for pharmacodynamics [11, 12]. In addition, animal pharmacodynamics experiment plus metabolic profiling with UHPLC-Q-TOF/MS was implemented to get a deeper understanding of the mechanism.

## 2. Materials and Methods

### 2.1. Filter of Effective Components about Chicory

Effective components of chicory were searched through Traditional Chinese Medicine Systems Pharmacology (TCMSP http://lsp.nwu.edu.cn/tcmsp.php) database. Alternative components needed to meet both oral bioavailability (OB) ≥ 30% and drug-likeness (DL) ≥ 0.18 conditions. Moreover, since TCMSP database did not include all chemical compositions, we also searched for latent components outside the database based on literatures from the past 10 years and then looked for homologous targets from the database. We took these chemical components and targets found in TCMSP database and references as a database in our research.

### 2.2. Network Construction

All target proteins have undergone gene names conversion in Uniprot (http://www.uniprot.org/) and were organized in Microsoft Excel 2016 spreadsheets. Component-target (C-T) network was used to identify possible components and their corresponding mechanisms pathway was visualized using Cytoscape 3.1.2.

### 2.3. Functional and Pathway Enrichment Analysis

Gene Ontology (GO) analysis and pathway analysis have become the main methods for functional studies. In present research, GO analysis involved three areas: the biological process (BP), cellular component (CC), and molecular function (MF) of the founded genes based on the gene ontology project. In the aspect of pathway analysis, the KEGG database contains information about interactions between molecules or genes [13]. In our research, the pathway enrichment analysis was predicted using ClueGO plugin for Cytoscape [14]; the* p* value <0.05 and enrichment count ≥2 were deemed as thresholds. Cluster Profiler R 3.4.3 was used to clarify functional and pathway enrichment analyses of massive genes acquired from previous database [15].

### 2.4. Drugs, Molding Agent, Chemicals, and Reagents

Chicory applied in our study was identified by Professor Yonghong Yan (Traditional Chinese Medicine Appraisal Teaching and Research Section of Beijing University of Chinese Medicine). Chicory was grinded into powder and weighed. The extracts were prepared with boiling water for 1 hour and extracted twice. Then a rotary evaporator was used to concentrate the solution after filtering and was deliquated to different volume with purified water [16].

Chemical characteristics obtained from this merchandise tested in our laboratory were the following: average molecular weight 1.38×10^3^; degree of polymerization range 2 to 30; and free glucose less than 5%. Fenofibrate tablets (0.1g) were acquired from Beijing Yimin Pharmaceutical Co., Ltd. The molding agent yeast extract was purchased from OXOID Co., Ltd, and mixed into the commercial feed formulation according to the weight ratio of 15 g.kg-1. Acetonitrile (HPLC grade) was acquired from Merck (Darmstadt, Germany), formic acid and methanol (HPLC grade) were obtained from Fisher Chemicals (Pittsburg, PA, USA), and water was purified by a Millipore's ultrapure water system. The reagents for the detection of serum uric acid (UA), total cholesterol (TC), triglyceride (TG), high density lipoprotein cholesterol (HDL-C), and low density lipoprotein cholesterol (LDL-C) were purchased from Jiancheng Biological Technology, Co., Ltd (Nanjing, China).

### 2.5. Quails, Diets, and Administration

All operations on quails were in conformity with the relevant provisions of the Animal Care and Ethics Committee in Beijing University of Chinese Medicine. The molding agent yeast extract is rich in purine [17]; thus, this modeling method is similar to the human high purine diet, which results in hyperuricemia triggered by purine nucleotide metabolic disorder. 32 male French quails acquired from a commercial rearing company (Beijing Deling Quail Farm) were randomly divided into the following four groups after one week of adaptive rearing: CON (control group fed with commercial quail feed formulation), MOD (model group fed a formulation with added yeast extract powder), FEN (fenofibrate treated group fed the same as the model group and treated with 30mg/kg fenofibrate water solution by intragastric administration with gavage needle ), CHI (chicory inulin treated group fed the same as the model group and treated with 10g/kg chicory inulin water solution by intragastric administration with gavage needle). Eight quails in each group were housed in 90×80×40 cm^3^ cages; temperature, humidity, and ventilating device are all in the standard environment (25±2°C temperature, 50-55% relative humidity and 12h photoperiod). All the quails were freely provided feed formulation and water during the experimental period. Feed formulation was obtained from Beijing Deling Quail Farm.

Feedstuff formulation was added twice per day and the surplus feedstuff would be weighed in each group next morning. Based on this, the average intake of food in each group was calculated at weekly intervals. The whole period of the experiment lasted 35 days.

### 2.6. Sample Collecting

Body weight was monitored weekly. Blood samples were gathered from the jugular veins after 12-hour fasting. These blood samples need to clot for 60 minutes at room temperature then centrifuge at 3500 rpm for 10 min. On the 30th day of the experiment, the metabolic cages were used to collect the stool samples (contained urine and fecal-mixture). Meantime, these quails were normal water supplied and fasted for 12 hours. Each stool was collected into the polypropylene tube; after adding 5 ml pure water vortex mixing was underway. After centrifugation, the supernatant was taken for use. The serum and stool were divided into two parts, respectively; one part was stored at -20°C for the detection of serum biochemical markers and the other part was stored at -80°C for UHPLC-Q-TOF/MS.

### 2.7. Sample Preparation for Metabolomic

100 *μ*L thawed serum and 300 *μ*L precooled methanol were transferred to a 1.5 mL polypropylene tube, respectively, and whirled more than 30s then placed in refrigerator at 4°C for more than 20 minutes to precipitate protein and other solids. After that, the sample was centrifuged at 12,000 rpm for 10 min at 4°C. Supernatants were gathered to filter through a syringe filter (0.22 *μ*m). The same method is for preparing stool samples.

### 2.8. Chromatography and Mass Spectrometry Condition

The Agilent 6550 iFunnel Q-TOF/MS system (Agilent Technologies, USA) was applied for chromatographic analysis. We had A ZORBOX RRHD C18 analytical column (100 mm × 2.1 mm., 1.7 *μ*m, Agilent Technologies, USA), and the column temperature was kept at 30°C and was served to chromatographic separation. The sample sequence was parallelism and 4 *μ*L of each sample was injected into the column. The optimal mobile phase was made up of 0.1% formic acid in water (solvent A) and 0.1% formic acid in acetonitrile (solvent B). The flow rate referenced to the literature was 0.3 mL/min with a gradient performed as below [18]: 0-1 min, 100% A; 1-9 min, 100 to 60% A; 9-19 min, 60 to 10% A; 19-21 min, 10 to 0% A; and 21-25 min, 0% A. Balance the column for 5 min in the initial mobile phase before injection. To ensure the stability and repeatability of the systems, a quality control (QC) sample was used to optimize the situation of UHPLC-Q-TOF/MS, since it contained majority of the information about the whole plasma and stool. All the samples were kept at 4°C during the experiment.

The mass spectrometry was performed by the Agilent 6550 Q-TOF/MS with an electrospray ionization source (ESI) in both positive and negative mode. The optimal conditions of analysis referenced to the literature were set as follows [18]: dry gas flow rate was 13 L/min; dry gas temperature was set at 200°C in negative ionization mode and 225°C in positive ionization mode. Sheath gas flow rate was 12 L/min and sheath gas temperature was 275°C. Electrospray capillary voltage was 3.5 kV in negative mode and 4.0 kV in positive mode. Nozzle voltage was 2.0 kV in negative mode and the same as in positive mode. Nebulizer pressure was set to 20 psig both in negative and positive mode.

### 2.9. Data Processing and Multivariate Data Analysis

The original data were preprocessed with Profinder software (Agilent, USA) for retention time correction and feature peak extraction then we obtained two-dimensional data sets including characteristic peak mass charge ratio, retention time, and peak intensity. MetaboAnalyst3.0 is used to filter and normalize the above data [19]. Then the data were imported to the SIMCA-P 13.0 version (Umetrics AB, Sweden), which was served to multivariate statistical analyses including principal component analysis (PCA) along with orthogonal partial least squares-discriminant analysis (OPLS-DA) [20]. Only variables with variable importance in projection (VIP) values* >*1 and |*p *(corr)| ≥ 0.5 were chosen and served to further data analysis.

### 2.10. Biomarker Identification and Metabolic Pathway Analysis

Components were selected as latent biomarkers with obvious changes among groups (*p* value* < *0.05 and folder change* > *2). They were identified by METLIN (https://metlin.scripps.edu/index.php) as well as KEGG (http://www.genome.jp/kegg/) database. To further appraise the potential metabolic pathways, the pathway analysis of these biomarkers was enforced by MetaboAnalyst 3.0 software (http://www.metaboanalyst.ca/) based on the pathway data library. Similarly, significant differences were considered when the* p* values < 0.05.

## 3. Results

### 3.1. Effective Components Screening

**Figure 1:**
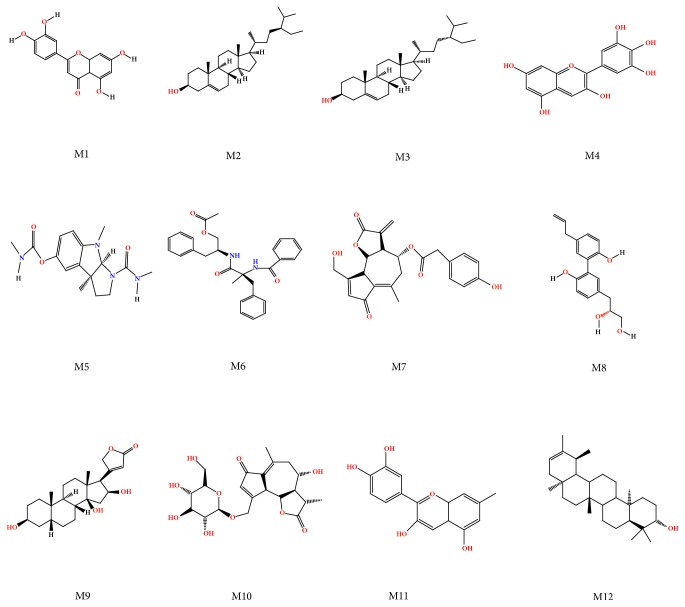
Chicory: 12 types of bone structure component.

By searching for TCMSP database, 54 effective components of chicory were obtained. On this basis, we picked out components that conformed to both OB ≥ 30% and DL ≥ 0.18 conditions. In addition, combined with literature screening, we acquired 12 related effective components. Some effective components with anti-inflammatory, antioxidation, and lipid metabolism regulation had been screened out, including luteolin, lactucopicrin, cyanidin, taraxasterol, and *β*-sitosterol. The chemical structures of these components are illustrated in Figure 1. Components represented by specific numbers in Table 1.

#### 3.1.1. Construction of Component-Target Prediction Network for Chicory

**Figure 2:**
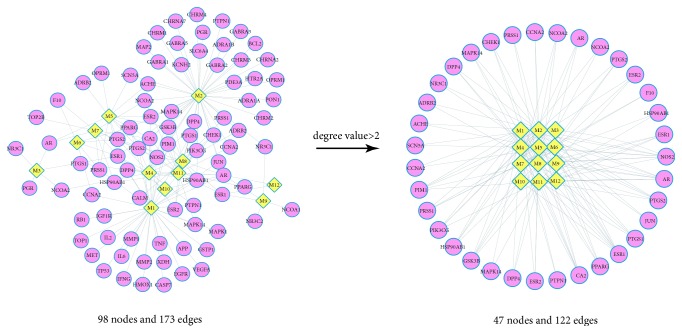
C-T network of chicory. A protein node and compound node are connected if the protein is targeted by the corresponding compound. The pink circles represent the chicory targets. The yellow diamonds represent the chicory components.

The mean degree value (the number of associated targets) of 69 alternative targets was 1, and by more than 2 times the mean degree value, we further screened potential targets and 25 targets were obtained. Simultaneously, the mean degree value of filtered components and targets was 1, and all the 12 components had a degree value >2, indicating that components play multiple therapeutic roles by regulating various targets. Specifically, 2 components, luteolin and beta-sitosterol, acted on 37 and 32 targets, respectively, meaning they are crucial active components of chicory because of their important positions in this network. Additionally, the results denoted that many targets were hit by multiple components in the C-T network (Figure 2).

#### 3.1.2. Enrichment Analysis of Prediction Targets

**Figure 3:**
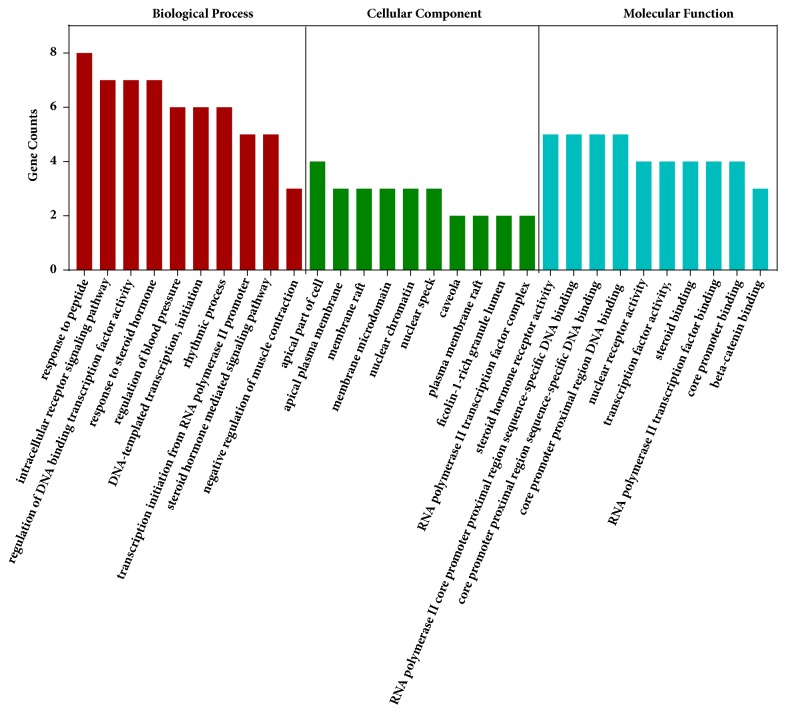
GO analysis of biological process (BP), cellular component (CC), and molecular function (MF). The top ten significant terms with the most gene counts are shown.

The comprehensive GO analysis was used to acquire a deeper understanding of the picked genes that were closely related to chicory. We used a total of 25 genes, which screened through the mean degree value, to enrich 573 GO terms (BP, 505; CC, 15 and MF, 53). Among the GO terms, the most remarkable biological process was intracellular receptor signaling pathway, in which 7 vitally enriched function clusters associated with this signaling pathway. The most prominent cellular component was apical part of cell, having 4 importantly enriched function clusters related to apical part of cell. And the most significant molecular function was steroid hormone receptor activity, which had 5 momentously enriched function clusters in connection with steroid hormone receptor activity (Figure 3).

**Figure 4:**
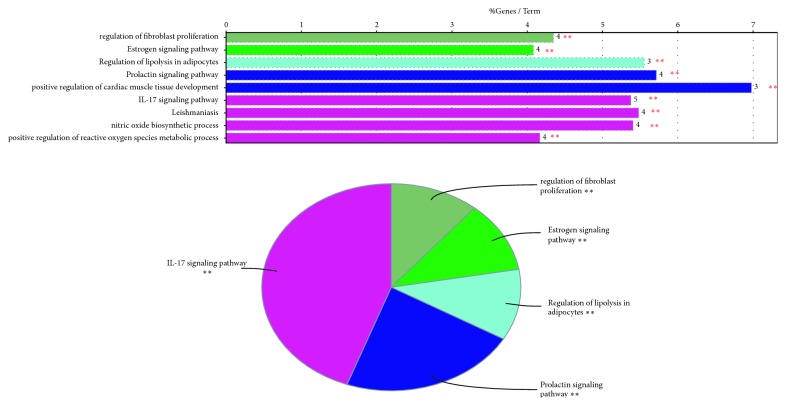
Significant enriched function pathways were represented by ClueGO analysis.

Using ClueGO to reveal biological processes about enrichment analysis of filtered targets, obvious differences could be found, among them IL-17 signaling pathway, regulation of lipolysis in adipocytes, estrogen signaling pathway, prolactin signaling pathway, regulation of fibroblast proliferation were particularly observed (Figure 4). Combined with the research findings of our previous laboratory and literature researches [10, 16, 21], we mainly concentrated the study of chicory on regulation of lipolysis in adipocytes.

### 3.2. Animal Experimental

#### 3.2.1. Pharmacodynamics Analysis of Chicory

**Figure 5:**
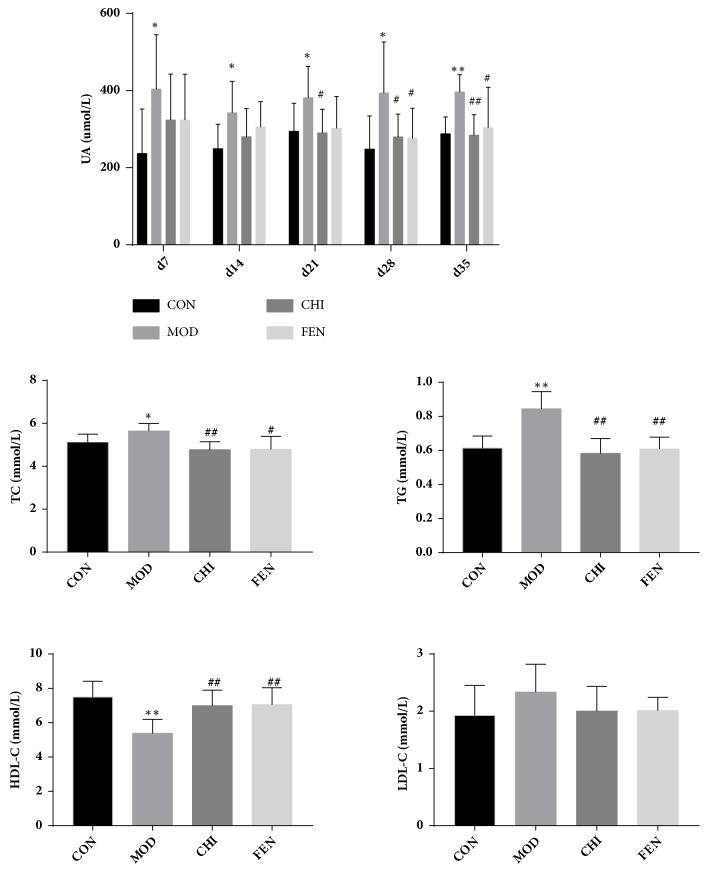
Reducing uric acid and antihyperlipidemia effects of chicory in hyperuricemia quails. Data were expressed as mean±S.E. ^*∗*^P < 0.05, ^*∗∗*^P < 0.01 vs. CON; #P<0.05, ##P<0.01 vs. MOD.

The levels of serum UA, TC, TG, HDL-C, and LDL-C in each group were shown in Figure 5. All values were shown as mean ± SD; T test and ANOVA were used to compare the differences variables among groups. Compared with CON, the level of UA in MOD increased significantly from day 7 and maintained to day 35 (P <0.05 and P < 0.01). The level of UA in the CHI and FEN gradually decreased and was statistically significant from day 21 to day 35 and day 28 to day 35, respectively (P <0.05 and P < 0.01). On day 35 of the experiment, we detected serum TC, TG, HDL-C, and LDL-C, respectively. Compared with CON, the levels of TC and TG in MOD elevated notable contrary to the HDL-C which decreased significantly (P <0.05). Compared with MOD, the levels of TC and TG in CHI and FEN decreased significantly (P < 0.01), and the levels of LDL-C in CHI and FEN reduced obviously. In addition, there was no difference in serum HDL-C among any of the groups.

#### 3.2.2. Multivariate Statistical Analysis and Potential Biomarkers

**Figure 6:**
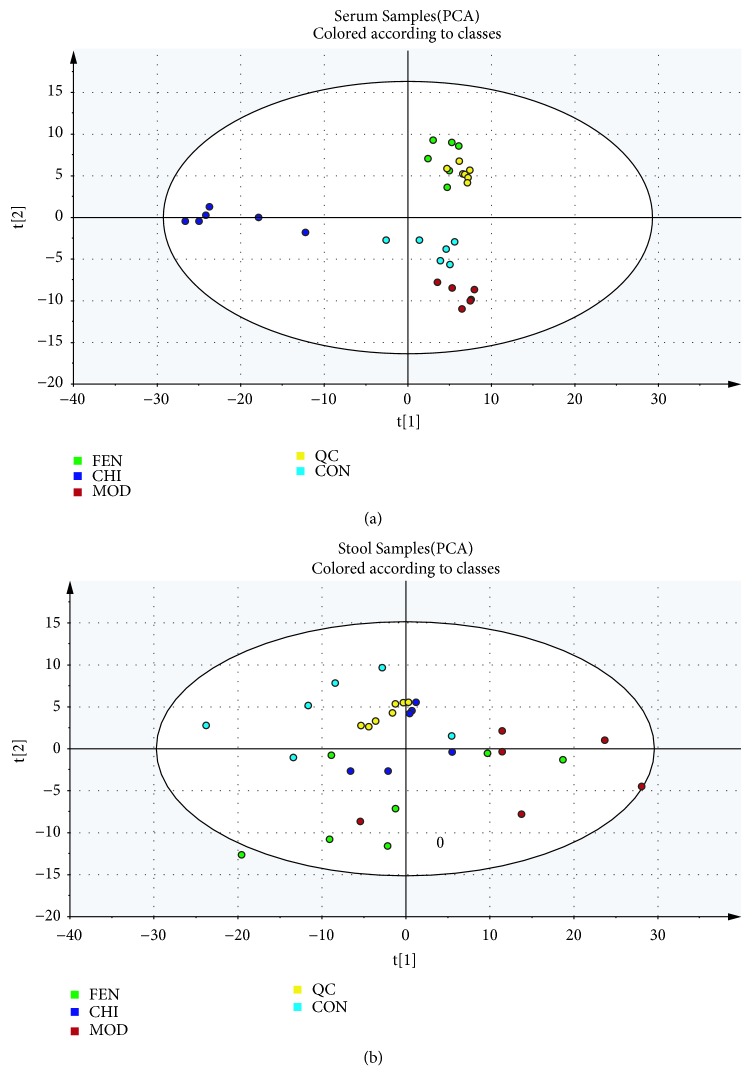
Principal component analysis (PCA) score of CON, MOD, FEN, and CHI groups in positive ESI modes. (a) serum samples and (b) stool samples.

The PCA score plot visually displayed the changes in the metabolic patterns of organisms among CON, MOD, FEN, and CHI groups. PCA analysis of serum and stool was shown in Figures 6(a) and 6(b), and in the positive ion common modes, QC samples clustered near the central position in the scoring matrix projection plot, indicating that the LC/MS system was stable throughout the analysis process. In addition, it was observed that in the positive ion mode, CON, MOD, FEN, and CHI groups could be better distinguished, indicating that the physiological metabolic environment among the four groups were changed. As shown in Figures 6(a) and 6(b), there were some slight overlaps between the control group and model group; however, the clustering significantly differed among the CON, MOD, FEN, and CHI groups in PCA. Further multivariate statistical analysis was necessary to distinguish the relationship among the CON, MOD, and CHI groups.

**Figure 7:**
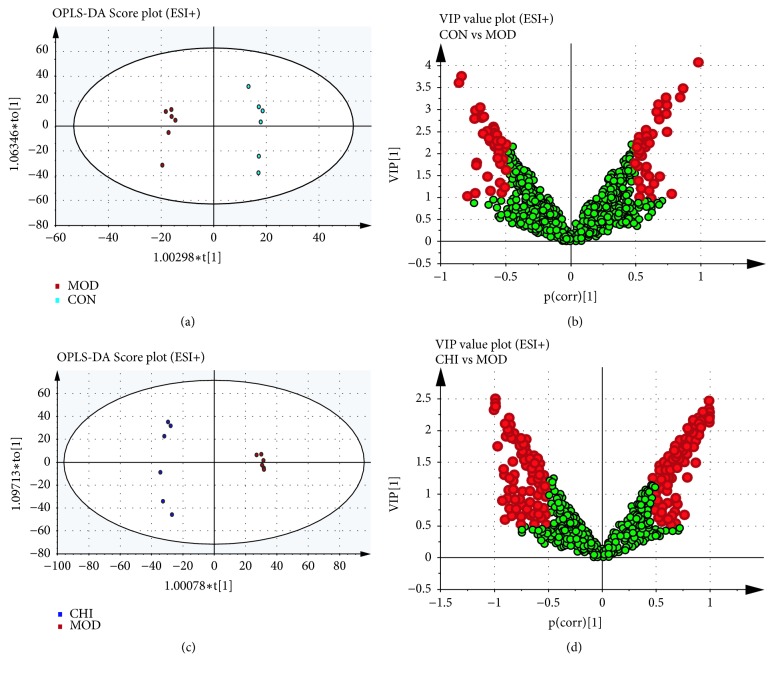
OPLS-DA analysis of serum samples data generated from CON, MOD, and CHI groups in the ESI^+^ mode. OPLS-DA score plots were the pair-wise comparisons between the CON and MOD (a) as well as CHI and MOD (c). VIP values of the OPLS-DA model were for the CON and MOD (b) along with CHI and MOD (d). The points in red indicate the identified biomarkers.

**Figure 8:**
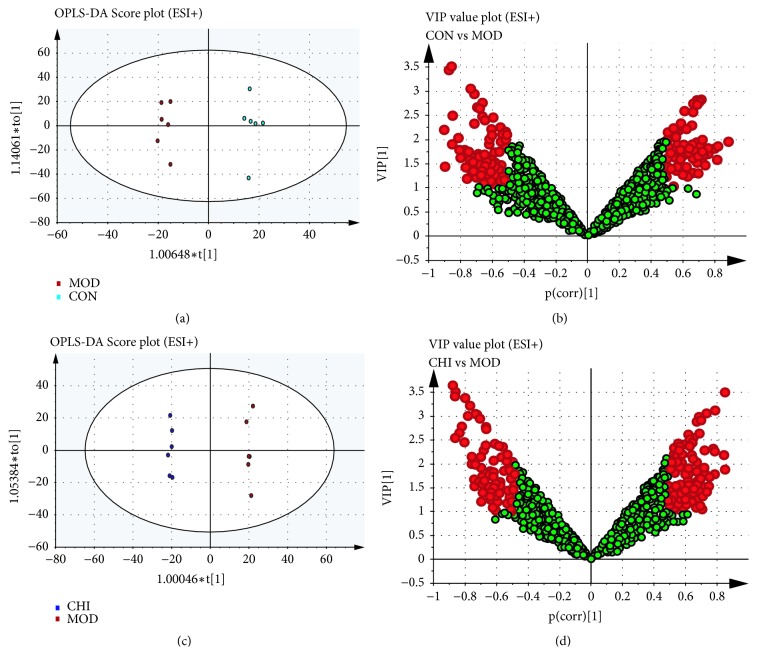
OPLS-DA analysis of stool samples data generated from CON, MOD, and CHI groups in the ESI^+^ mode. OPLS-DA score plots were the pair-wise comparisons between the CON and MOD (a) as well as CHI and MOD (c). VIP values of the OPLS-DA model were for the CON and MOD (b) along with CHI and MOD (d). The points in red indicate the identified biomarkers.

OPLS-DA was served to investigate potential biomarkers in different groups. The results of OPLS-DA model originated from data of ESI^+^ analysis about the serum and stool samples were shown in Figures 7 and 8. As shown in OPLS-DA score plots, there was a distinguished classification among the clustering of the CON, MOD, and CHI groups suggesting that metabolic profiles significantly changed in the 3 groups. There were a total of 21+127 endogenous biomarkers screened out. Similarly, 23+228 endogenous biomarkers were screened in ESI^+^ analysis suggesting that metabolic profiles significantly changed in 3 groups. Commonly, the R2X and Q2X provide an estimate of predictive ability in OPLS-DA model. In the ESI+ model, the parameters about serum for classification from the software were R2X =0.388 and Q2X= 0.288, and for stool samples, in the ESI^+^ model, R2X= 0.627 and Q2X= 0.269, respectively, indicating that the OPLS-DA model was well established. VIP>1 or |*p *(corr)| ≥ 0.5 were selected difference variables as potential candidate markers, which might be the key metabolites of inulin acted on hyperlipidemia quail.

#### 3.2.3. Identification of Potential Metabolites Pathway of Chicory

**Figure 9:**
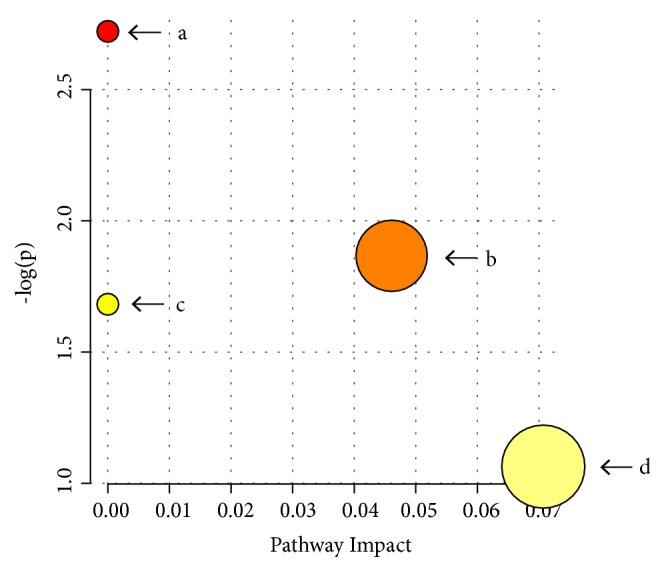
Summary of pathway analysis in serum samples: (a) glycosylphosphatidylinositol (GPI)-anchor biosynthesis; (b) inositol phosphate metabolism; (c) glycerophospholipid metabolism; and (d) steroid hormone biosynthesis.

Based on the above research, in the metabolomic of serum and stool samples, 22 components were identified as endogenous biomarkers via combining all the markers of positive ions, and by comparing the exact mass ratio of KEGG to the METLIN database, 9 and 94 biomarkers were further screened through KEGG pathway analysis, respectively, which were screened into the MetaboAnalyst software for enrichment pathway analysis, then 4 and 15 metabolic pathways were screened, respectively, in the serum and stool samples. According to the results of metabolomic, we concluded that the 4 potential mutual pathways where chicory could act on the HUA quails model were finally obtained. As shown in Figure 9, the common pathways of steroid hormone biosynthesis, inositol phosphate metabolism, glycerophospholipid metabolism, and glycosylphosphatidylinositol (GPI)-anchor biosynthesis were selected. Combined with previous predictions and experimental results, we will focus on discussing glycerophospholipid metabolism.

## 4. Discussion

Hyperuricemia is positively correlated with hyperlipidemia, diabetes, atherosclerosis, obesity, and other metabolic disorders. Related studies have indicated that hyperlipidemia and obesity are often accompanied by HUA. Chicory, which is a cholagogic and diuretic agent in Uighur folk medicine, is widely used as the forage, leafy vegetable, crude materials for sugar and coffee substitute materials. The literature showed that the pharmacological functions of chicory include antioxidant, anti-inflammatory, antihyperlipidemia, and hypoglycemic effects [22, 23]. In addition, chicory was found to significantly reduce the level of serum uric acid and regulate lipid metabolism, such as inhibiting the activities of uricopoiesis metabolic enzymes of xanthine oxidase [24] and upregulating intestinal ABCG2 mRNA and protein as well as the renal OAT3 mRNA expressions to promote excretion of uric acid [9]. But what are the potential components and possible pharmacodynamic mechanism of chicory regarding the effect of antihyperuricemia?

In recent years, network pharmacology has provided a new research method for scientific research in the field of traditional Chinese medicine, which can offer the prediction for drug component and its corresponding target research through network construction based on big data. It helps us to find the possible ways of action of the drug through enrichment analysis, bringing hints to subsequent research to make later study more purposeful. In this study, we constructed a total of 12 effective components and 25 potential targets by constructing a C-T prediction network. Flavonoid and terpenoids were the main components, and the prediction results were consistent with experiments [25]. Among these components, *β*-sitosterol had the effect of inhibiting lipid peroxidation, regulating lipid metabolism, and preventing atherosclerosis [26]. Luteolin depressed insulin resistance [27]. In addition, psi-taraxasterol also have the effect of reducing lipid [28], and delphinidin can reduce intracellular lipid accumulation in vitro markedly through the modulation of the gene expressions related lipid metabolism [29]. These predicted components were consistent with the previous researches involved in the efficacy of chicory basically [30–32]. Through functional annotation and enrichment analysis of the targets corresponding to the components, we have obtained several major metabolic pathways. Based on these studies we can see that the results of our predictions through network pharmacology were reasonable.

Based on the previous research on chicory in our laboratory [16, 21], in this research, we selected quail as the experimental hyperuricemia animal model. Through the serum biochemical indexes in animal experiment showed that the model of quails developing HUA were successfully fed with purine-rich diet, and chicory had obvious effect of reducing uric acid and antihyperlipidemia. This was consistent with the results of the previous bioinformatics predictions that chicory exerted a regulation of lipolysis in adipocytes. Moreover, in many studies, chicory had improved significantly in the treatment of metabolic diseases such as diabetes and atherosclerosis [33, 34]. However, in terms of pharmacological mechanism, few correlation researches have been involved in metabolomic research, and which metabolic pathway of chicory exerts pharmacodynamic is still unclear. Metabolomics, a comprehensive analysis of endogenous metabolites of the synthetic biological system, is a very important technique to explore the potential biomarkers related to the diagnosis of diseases or the efficacy of drugs [35]. Here, our LC/MS based metabolomic research on the serum and stool samples reveals which metabolic pathway and metabolites have effect on the efficacy of chicory.

In multivariate statistical analysis, CHI was far away from MOD and even away from CON; we consider that it may be related to the dosage of chicory, or there were other potential pharmacological mechanisms of chicory; further studies are still necessary to explore. Subsequently, in the analysis of serum and stool samples metabolomics, combined with the above results, we focus on discussion of glycerophospholipid metabolism.

In the pathway of glycerophospholipid metabolism, stearic acid, 1-acyl-sn-glycerol 3-phosphate, 1-acylglycerophosphoinositol, and 1-acyl-sn-glycero-3-phosphoglycerol were upregulated in MOD more than CON, and hexadecanoic acid, l-palmitoylcarnitine, and nonanedioic acid were downregulated in MOD. Chicory adjusts glycerol phospholipid metabolism by reversing these metabolites. Combined with the results of this study, it is suggested that abnormal metabolism of glycerophospholipid may be one of the metabolic pathways involved in the pathogenesis of HUA. As we all know, the state of HUA has the effect of promoting oxidation as well as inducing translocation of the nicotinamide adenine dinucleotide phosphate (NADPH) oxidase subunit in mitochondrion. This results in increased mitochondrial oxidative stress and mitochondrial dysfunction along with release of citrate to the cytoplasm for lipid and triglyceride synthesis [36]. In addition, xanthine oxidase catalyzed the conversion of hypoxanthine or xanthine to uric acid accompanied by a large number of free radical production [37, 38]. Furthermore, studies have shown that urate crystallization can inhibit the reduction of fatty acid oxidation and triglyceride accumulation by AMP kinase [39, 40].

Fatty acids such as stearic acid, and 1-acyl-sn-glycerol 3-phosphate are mainly involved in the synthesis of fatty acids, the extension of fatty acids in mitochondria, fatty acid metabolism, and the synthesis of unsaturated fatty acids [41, 42]. These metabolites disorders suggested that there might be disorders of fatty acid metabolism in HUA. In addition, related studies had shown that stearic acid can be used as a metabolite spectrum of coronary atherosclerotic heart disease [43]. Excessive intake of saturated fatty acids is a risk factor for hyperinsulinemia, especially hexadecanoic acid, which could promote the occurrence of type 2 diabetes [44, 45]. These studies indicated that HUA was closely related to coronary heart disease, diabetes, hypertension and so on, which was reflected in the disorder of lipid metabolism. Chicory exerted the dual effects of reducing uric acid and lipid-lowering by adjusting these lipid and purine metabolic enzymes, especially stearic acid, regulating the pathological state of model animals.

In summary, chicory is beneficial to regulate the disorder of lipid metabolism for hyperuricemia. In this study, we chose quail as animal model innovatively and explored the treatment of hyperuricemia with chicory in multiple methods to provide reference for the study of hyperuricemia. In addition, we have to point out that we need to further build the target-disease (T-D) network to discuss the prediction about disease in the treatment of chicory. Moreover, only LC/MS was used to analyze samples and detected potential biological markers with biological significance. It could be seen that our detection method was simple and still needed further comprehensive research to combine with GC-MS, NMR, etc. Meanwhile, more researches needed to combine with molecular biology experiments to elucidate the pharmacological mechanisms of chicory lowering uric acid and antihyperlipidemia effects systematically.

## 5. Conclusion

In conclusion, firstly, the present study predicted the effective components and targets of chicory via bioinformatics analysis, which acquired 12 related effective components along with 25 corresponding targets, and these genes were enriched in regulation of lipolysis in adipocytes. Secondly, animal pharmacodynamics experiment combined with metabolomic profiling with UHPLC-Q-TOF/MS methods, and 9 and 94 potential biomarkers were screened in the serum and stool samples, respectively, and enriched in the corresponding metabolic pathways. Finally, under our experimental conditions, it is suggested that chicory may play a role in lowering uric acid and antihyperlipidemia by adjusting lipid metabolism. Our research not only supplies new method for the search for potential active components but also provides comprehensive analysis to interpret the underlying therapeutic effects of chicory in diet-induced HUA. However, it is important to note that bioinformatic and metabolomic analyses do not provide any conclusive evidence, but rather provide hypothesis and references for future studies, and we need to confirm these conclusions.

## Figures and Tables

**Table 1:** Components represented by specific numbers.

Number	Component	Molecular Formula	OB%	DL
M1	Luteolin	C_15_H_10_O_6_	36.16	0.25
M2	Beta-Sitosterol	C_29_H_50_O	36.91	0.75
M3	Poriferast-5-en-3beta-ol	C_29_H_50_O	36.91	0.75
M4	Delphinidin	C_15_H_11_O_7_	40.63	0.28
M5	Eseramine	C_16_H_22_N_4_O_3_	45.89	0.31
M6	2S,2′S-Aurantiamide acetate	C_27_H_28_N_2_O_4_	39.18	0.54
M7	Lactucopicrin	C_23_H_19_O_7_	95.31	0.71
M8	Magnolignan A	C_18_H_20_O_4_	32.21	0.2
M9	Gitoxigenin	C_23_H_34_O_5_	43.93	0.75
M10	Cichorioside B	C_21_H_28_O_10_	32.05	0.8
M11	Cyanidin 3-glucoside_qt	C_16_H_13_O_5_	58.99	0.24
M12	Psi-taraxasterol	C_30_H_50_O	39.75	0.76

## Data Availability

Our data used to support the findings of this study are available from the corresponding author upon request.
